# Autosomal Dominant Pseudohypoaldosteronism Type 1 in a Newborn With Failure to Thrive

**DOI:** 10.7759/cureus.59356

**Published:** 2024-04-30

**Authors:** Sunil Krishna, Mary Augustian

**Affiliations:** 1 Pediatrics/Neonatology, University of Illinois, Rockford, USA; 2 Pediatric Medicine, Mercyhealth System, Janesville, USA

**Keywords:** genetic variant, failure-to-thrive, newborn infant, autosomal dominant disorder, pseudohypoaldosteronism type 1

## Abstract

Pseudohypoaldosteronism type 1 is a rare genetic disorder characterized by salt wasting and resistance to mineralocorticoids due to mutations in the *NR3C2* gene which codes for the aldosterone receptor proteins in the kidneys. This case study involves an infant who presented with poor growth and significant hyponatremia. There was improvement in growth and correction of hyponatremia with sodium supplementation, later found to carry a new genetic variant causing autosomal dominant pseudohypoaldosteronism type 1. A 14-day-old newborn presented with failure to thrive, severe hyponatremia, mild hyperkalemia, and metabolic acidosis. The electrolyte abnormalities were corrected with intravenous fluid and sodium supplementation. Continued oral sodium supplementation led to improved weight gain. Clinical suspicion and subsequent diagnostic testing led to a diagnosis of the autosomal dominant renal form of pseudohypoaldosteronism type 1. Genetic testing revealed a novel mutation on the *NR3C2* gene, c.556_557del (p.Met186Valfs*3). The baby was discharged home on supplemental sodium and high-calorie formula for catch-up growth. Outpatient follow-up is ongoing.

## Introduction

Pseudohypoaldosteronism encompasses a diverse range of rare conditions characterized by resistance of the end organs to aldosterone which in turn can lead to hyponatremia, hyperkalemia, and metabolic acidosis [1]. Cheek and Perry originally documented this in 1958 [2]. There are two distinct types of pseudohypoaldosteronism, namely type 1 (PHA-I) and type 2 (PHA-II). PHA-I can be inherited through autosomal dominant (PHA1A) or autosomal recessive patterns (PHA1B) [3]. PHA1B (MIM #264350) is caused by loss-of-function mutations in the epithelial sodium channel. This variant is identified by extensive salt depletion affecting various organs including the lung, kidney, colon, and sweat and salivary glands [4]. By contrast, PHA1A (MIM #177735) is linked to heterozygous mutations on the *NR3C2 *gene on chromosome 4q31, coding for the aldosterone receptor, affecting the transport of sodium within the principal cells of the kidney [5]. PHA-I commonly appears during infancy, showing symptoms such as salt loss, poor growth, high potassium levels, and acidosis, which are similar to those seen in infants with congenital adrenal hyperplasia. The reduced sodium reabsorption by epithelial cells in the connecting segments and cortical collecting duct leads to volume depletion. Consequently, this reduction diminishes the usual electrochemical gradient that promotes the secretion of potassium and hydrogen ions [6]. In this report, we present a case of PHA1A with a novel variant of NR3C2 in an infant with failure to thrive and hyponatremia.

## Case presentation

We received a 14-day-old female infant from a level-II unit at our regional perinatal center because of a 12% weight loss from birth, vomiting after feeds, and mild abdominal distension. The infant was born to a 20-year-old, G1P0, mother by C-section for breech presentation at 36 weeks gestation with a birth weight of 3100 g (38th percentile, WHO growth chart for girls). The mother did not receive adequate prenatal care, with an unknown blood type, Group B Streptococcus status, Rapid Plasma Reagin status, and rubella immunity. She tested negative for HIV and hepatitis B. Mother has a history of septate uterus and Von Willebrand disease. No family history of pregnancy losses or neonatal deaths. The infant's mother was advised to feed 2 oz. of Enfamil formula every 2 hours, but the baby typically only consumed 1.0 oz. with occasional episodes of non-bilious vomiting after feeds. The infant's weight on admission at 14 days of age was 2720 g. The infant's physical examination revealed low-set cupped ears, bilateral epicanthal folds, mild jaundice, hypotonia, and abdominal distension. The rest of the systemic examination was unremarkable with normal female genitalia.

Initial investigations ruled out sepsis but revealed hyponatremia with a plasma sodium of 126 mmol/L, potassium levels of 5.8 mmol/L, and mild metabolic acidosis with a bicarbonate level of 18 mmol/L. Initially, the infant received intravenous sodium correction, followed by an increase in sodium supplementation over the first ten days of admission (up to 8 mEq/kg/day), with a slow correction of plasma sodium levels to 136 mmol/L. The infant also began to gain weight appropriately as the plasma sodium levels normalized. Plasma potassium levels were mildly elevated at 5.7 to 6.3 mmol/L but gradually normalized over time. Elevated random urine sodium levels were observed despite the presence of hyponatremia, with initial results showing 45 mmol/L and subsequent levels at 187 mmol/L. Additionally, the urine potassium level was measured at 60 mmol/L, while specific gravity stood at 1.013 and pH at 6.0. Osmolality was determined to be 466 mOsm/kg, as shown in Table 1.

**Table 1:** The Biochemical Data of the Patient BUN: blood urea nitrogen; ACTH: adrenocorticotropic hormone

Parameters (Reference Range)	Day of Life 14	Day of Life 20	Day of Discharge
Serum sodium (132-142mmol/L)	126	136	136
Serum potassium (3.3-5.1 mmol/L)	5.8	5.4	5.9
Serum chloride (98-107 mmol/L)	93	104	103
Serum bicarbonate (22-29 mmol/L)	18	21	20
Serum BUN (3-12 mg/dL)	20	11	12
Serum creatinine (0.1-0.3 mg/dL)	0.4	0.3	0.3
Urine sodium (< 20 mmol/L)	45	187	-
Urine potassium (<20 mmol/L)	60	57	-
Urine osmolality (50-1200 mOsm/kg)	466	-	-
ACTH (7.2-63.3 pg/mL)	-	21.3	-
Cortisol (6-18.4 mcg/dL)	-	20	-
Aldosterone (5-175 ng/dL)	-	645	-
Hydroxyprogesterone 17OH (13-106 ng/dL)	-	20	-

After an endocrinology consultation, a normal adrenocorticotropic hormone (ACTH) level was obtained, followed by a normal result on the ACTH stimulation test. The blood aldosterone level was elevated at 645 ng/dL, whereas the hydroxyprogesterone 17OH level remained within the normal range. Given the persistent requirement for elevated sodium intake to restore regular plasma sodium levels and high aldosterone concentrations, a diagnosis of pseudohypoaldosteronism was considered. Genetic testing through the Invitae renal tubular disorders panel revealed a new heterozygous pathogenic variant, NR3C2, Exon 2, c.556_557del (p.Met186Valfs*3). This variant is consistent with a diagnosis of autosomal dominant pseudohypoaldosteronism type 1 (PHA1A). The test also showed an additional variant of uncertain significance, as shown in Table 2.

**Table 2:** Invitae Renal Tubular Disorders Panel

Gene	Variant	Zygosity	Variant Classification
NR3C2	c.556_557del (p.Met186Valfs*3)	Heterozygous	Pathogenic
GATM	c.701A>G (p.Asp234Gly)	Heterozygous	Uncertain significance

In light of the mild dysmorphic facial features, a metabolic workup was performed, which included tests for ammonia, lactic acid, pyruvate, plasma amino acids, urine organic acids, acyl-carnitine profile, and carnitine free/total levels. Additionally, a comprehensive fatty acid profile was conducted and all results were found to be normal. Furthermore, a whole-genome chromosome single‐nucleotide polymorphism (SNP) microarray analysis also yielded normal findings. In addition to these tests, a cranial ultrasound, echocardiogram, and renal ultrasound showed no abnormalities. At 35 days of age, the infant was discharged home with sodium supplementation at a dose of 6.5 mEq/kg/day and close monitoring of plasma sodium levels. The infant weighed 3100 g at discharge (5th percentile, WHO growth chart for girls). At three months old, the baby is growing well (25th percentile) on sodium supplementation and maintaining normal plasma sodium and potassium levels.

## Discussion

Autosomal dominant PHA1A is a rare disorder, with an incidence of 1 in 80,000. The control of sodium reabsorption and potassium excretion in the distal tubular nephrons is primarily mediated by aldosterone. This steroid hormone is synthesized in the adrenal gland zona glomerulosa in response to renin stimulation via angiotensin II. Aldosterone exerts its effects by binding to its receptor, the aldosterone receptor [7]. The aldosterone receptor is encoded by the *NR3C2 *gene located on chromosome 4q31. Heterozygous mutations in this gene lead to the unresponsiveness of these receptors to aldosterone, resulting in decreased absorption of sodium and increased excretion of potassium within the principal cells of the distal tubules in the kidney [8]. PHA1A is often identified when infants are around 2 to 4 weeks old, and in some cases within the first 10 days, due to inadequate weight gain or weight loss [3]. It is also usually associated with hyponatremia, hyperkalemia, and metabolic acidosis [9]. Aldosterone resistance in PHA1A is limited to the kidneys, and typically, there is no need for salt supplementation by the age of 1-3 years. The long-term outlook for normal kidney function, intelligence, and life expectancy is good. Rarely PHA1A can be fatal in the newborn if not diagnosed early and treated with salt supplementation [10]. The differential diagnosis may include congenital adrenal hyperplasia, hypoadrenalism, congenital adrenal hypoplasia, isolated aldosterone deficiency, and nephrotoxic medications such as angiotensin-converting enzyme (ACE) inhibitors and nonsteroidal anti-inflammatory drugs [11, 12]. As shown in our patient, low plasma sodium, hyperkalemia, relatively high urine sodium, and elevated aldosterone levels are consistent with a diagnosis of pseudohypoaldosteronism. The new and pathogenic heterozygous variant identified in our case, NR3C2, Exon 2, c.556_557del (p.Met186Valfs*3), creates a premature translational stop signal (p.Met186Valfs*3) in the *NR3C2 *gene leading to absent or disrupted protein function. This variant has not been reported in population databases (gnomAD) or in the literature for individuals affected with NR3C2-related conditions. It is essential to raise awareness among healthcare providers about the clinical features and diagnostic approach for PHA1A. Early recognition and intervention can significantly impact the long-term outcome for affected infants.

## Conclusions

This case underscores the importance of considering rare genetic disorders like autosomal dominant pseudohypoaldosteronism type 1 in the differential diagnosis of infants presenting with failure to thrive and electrolyte imbalances. Through this report, we aim to contribute to the growing body of knowledge surrounding this rare genetic disorder and emphasize the need for early detection and appropriate management to optimize the outcomes for affected infants.
